# Hépatite virale B lors d´une campagne de dépistage en population générale au Bénin: séroprévalence et facteurs associés

**DOI:** 10.11604/pamj.2020.37.247.26070

**Published:** 2020-11-18

**Authors:** Aboudou Raïmi Kpossou, Moussiliou Noel Paraiso, Comlan N’déhougbèa Sokpon, Khadidjatou Saké Alassan, Rodolph Koffi Vignon, René Kpemahouton Keke, Cédric Bigot, Camille Domonhédo, Edmond Sossa Gbédo, Jean Séhonou, Nicolas Kodjoh, Hervé Lawin

**Affiliations:** 1 Faculté des Sciences de la Santé, Université d´Abomey-Calavi, Cotonou, Bénin,; 2 Centre National Hospitalier et Universitaire Hubert Koutoukou Maga de Cotonou, Cotonou, Bénin,; 3 Institut Régional de Santé Publique (IRSP), Ouidah, Bénin,; 4 Service de Médecine Interne, Centre Hospitalier Universitaire Départemental Borgou-Alibori, Parakou, Bénin,; 5 Laboratoire National de Référence du Programme Santé de Lutte contre le Sida, Cotonou, Bénin,; 6 Rotary International, Cotonou, Bénin,; 7 Programme National de Lutte contre les Hépatites (PNLH), Cotonou, Bénin,; 8 Unité d'Enseignement et de Recherche en Santé au Travail et Environnement, Faculté des Sciences de la Santé, Université d´Abomey-Calavi, Cotonou, Bénin,; 9 Chaire EcoSanté, Faculté des Sciences de la Santé, Université d´Abomey-Calavi, Cotonou, Bénin

**Keywords:** Virus de l’hépatite B, profil épidémiologique, Bénin, Hepatitis B virus, epidemiological profile, Benin

## Abstract

**Introduction:**

l´hépatite B est un problème mondial de santé publique. Le but de cette étude était de déterminer la prévalence et les facteurs associés au portage du virus de l´hépatite B (VHB) à l´occasion d´un dépistage de masse dans plusieurs localités réparties sur le territoire béninois.

**Méthodes:**

il s´agissait d´une étude transversale descriptive et analytique avec une collecte prospective de données. Cette collecte avait eu lieu du 20 au 28 juillet 2019 à l´occasion d´un dépistage volontaire gratuit de l´hépatite B organisé au Bénin par les Club Rotary sur 23 sites répartis sur tout le territoire national. Un test rapide d´orientation diagnostique avait été utilisé pour la détection de l´Antigène HBs.

**Résultats:**

en tout, 9035 volontaires étaient inclus, pour la plupart de nationalité Béninoise (99%), avec un âge médian de 27 ans, dont 51,1% de célibataires et principalement des écoliers, élèves ou étudiants (37,9%). La séroprévalence du VHB était de 6% (545/9035) [IC95%, 5%-6, 5%]. Les facteurs associés au portage de l´Ag HBs étaient, en analyse univariée: l´âge, le sexe, le statut matrimonial, le niveau d´étude, la profession et les antécédents de diabète, de scarification et d´hépatite C; et en analyse multivariée: le sexe, l´âge, et le niveau d´étude.

**Conclusion:**

la séroprévalence du VHB est de 6% dans la population étudiée. L´infection par le VHB semble toucher volontiers les sujets de sexe masculin, âgés de plus de 17 ans, et ayant un niveau d´étude secondaire.

## Abstract (en)

**Introduction:**

hepatitis B is a global public health problem. The purpose of this study was to determine the prevalence of hepatitis B virus (HBV) and its associated factors based on data from a mass screening in several localities across Benin.

**Method:**

we conducted a cross-sectional descriptive and analytical study with prospective data collection. Data collection was carried out during voluntary free hepatitis B screening organized by the Rotary Club in 23 localities across Benin from the 20^th^ to 28^th^ July 2019. Rapid test for diagnostic orientation was used for the detection of HBs antigen.

**Results:**

a total of 9035 volunteers were included in the study. They were mostly of Benin origin (99%), with a mean age of 27 years; 51.1% of them were singles, mainly schoolchildren, pupils or students (37.9%). HBV seroprevalence was 6% (545/9035) [95%CI=5.5%-6.5%]. In univariate analysis, factors associated with HBs Ag colonization were: age, sex, marital status, education level, occupation and a history of diabetes, scarification and hepatitis C, while in multivariate analysis they were: sex, age and education level.

**Conclusion:**

HBV seroprevalence is 6% in the study population. HBV infection mainly affects male subjects over 17 years of age with secondary-level education.

## Introduction

L´hépatite B est une inflammation du foie causée par le virus de l´hépatite B (VHB). Ce virus entraîne des hépatites aiguës qui évoluent vers des hépatites chroniques dans 5% chez l´adulte, et jusqu´à 90% chez l´enfant [1,2]. L´hépatite chronique B est l´une des principales causes de cirrhose et de cancer primitif du foie en Afrique subsaharienne. Dans les pays à forte endémicité pour l´hépatite virale B (HVB), le VHB se transmet communément par voie verticale, de la mère au nouveau-né essentiellement au moment de la naissance, et par voie horizontale, aux sujets contacts vivant dans l´entourage des sujets infectés, particulièrement dans la période périnatale [3]. Les autres modes de contamination sont l´exposition percutanée ou muqueuse à du sang infecté, ou à d´autres sécrétions biologiques, et la transmission sexuelle favorisée par les comportements à risque (multipartenariat sexuel, polygamie, homosexualité masculine, mode de circoncision...) [3,4].

L´HVB est un problème majeur de santé publique. En effet, à l´échelle mondiale, l´Organisation Mondiale de la Santé (OMS) estime que 257 millions d´individus (soit 3,5%) sont porteurs chroniques de l´hépatite B [5]. L´OMS estime qu´en 2015, environ 887 000 personnes sont décédées d´une hépatite B, le plus souvent des suites d´une cirrhose ou d´un cancer primaire du foie. L´hépatite B est une affection ubiquitaire, le plus souvent asymptomatique. Toutefois, les Régions de l´OMS les plus affectées sont l´Afrique et le Pacifique Ouest, avec une prévalence estimée en 2015 à 6,1% et 6,2%, respectivement [5]. Au Bénin, sa prévalence nationale n´est pas connue, elle a été estimée à 9,9% d´après une enquête en 2013 chez les nouveaux donneurs de sang [6]. Des prévalences plus élevées ont été rapportées dans certaines populations: 15,5% chez les femmes enceintes en 2014 à Tanguiéta [7] et 11,7% chez les personnes privées de liberté en 2015 dans quatre principales villes du Bénin [8]. D´après une étude dans la Clinique Universitaire d´Hépato-gastroentérologie du Centre National Hospitalier Universitaire - Hubert Koutoukou Maga (CNHU-HKM) de Cotonou, l´hépatite B touchait plus les adultes jeunes (39,7 ans d´âge moyen), volontiers de sexe masculin (sex-ratio=2,5) [9].

Actuellement, les traitements utilisés contre l´hépatite B ne permettent pas une guérison mais vise à stopper la réplication virale afin de réduire le risque de cirrhose et de carcinome hépatocellulaire. Cependant, il existe un vaccin très efficace pour prévenir l´hépatite B. Au Bénin, depuis 2002, ce vaccin a été administré aux nourrissons dans le cadre du Programme Elargi de Vaccination (PEV) à partir de 6 semaines de vie [6]. Toutefois, l´OMS recommande le vaccin contre l´hépatite B soit plutôt administré chez tous les nouveau-nés dès les 24 premières heures de vie [6].

Afin d´appuyer les pays dans leur progression vers les cibles mondiales d´élimination de l´hépatite dans le cadre du Programme de développement durable à l´horizon 2030, l´OMS s´efforce entre autres de sensibiliser sur cette affection. Pour la Journée mondiale de l´hépatite 2019, elle a choisi comme thème principal « Investir dans l´élimination de l´hépatite ». C´est ainsi qu´est née la volonté manifeste des Clubs Rotary du Bénin à accompagner le Rotary International à faire de la lutte contre les hépatites un combat noble pour le bien-être des populations et de participer à la semaine panafricaine de lutte contre les hépatites. Dans ce cadre, une campagne de dépistage gratuit des hépatites virales B et C avait été organisée dans différentes localités du Bénin pour permettre de prendre connaissance de l´ampleur de l´infection et planifier d´autres actions afin de parvenir à Zéro cas d´Hépatite au Bénin. Nous avons donc profité de cette opportunité pour réaliser ce travail dont l´objectif était de déterminer la prévalence de l´hépatite B et les facteurs associés à cette infection chez les volontaires dépistés.

## Méthodes

**Type d´étude**: il s´agissait d´une étude transversale descriptive et analytique, avec une collecte prospective de données.

**Contexte**: les données de cette étude sont issues d´une séance de tests volontaires, organisée par les clubs Rotary du Bénin du 20 au 28 juillet 2019, et qui avait concerné des hommes et des femmes de tous les âges. C´était une activité relative à l´Action internationale de tous les clubs Rotary du monde. Elle s´intégrait dans l´axe stratégique n°2 du Rotary International à savoir: la prévention et le traitement des maladies. L´action avait porté spécifiquement sur le dépistage volontaire des hépatites B et C prévu dans un cadre intitulé: hépatite Zéro-Bénin.

**Figure 1:**
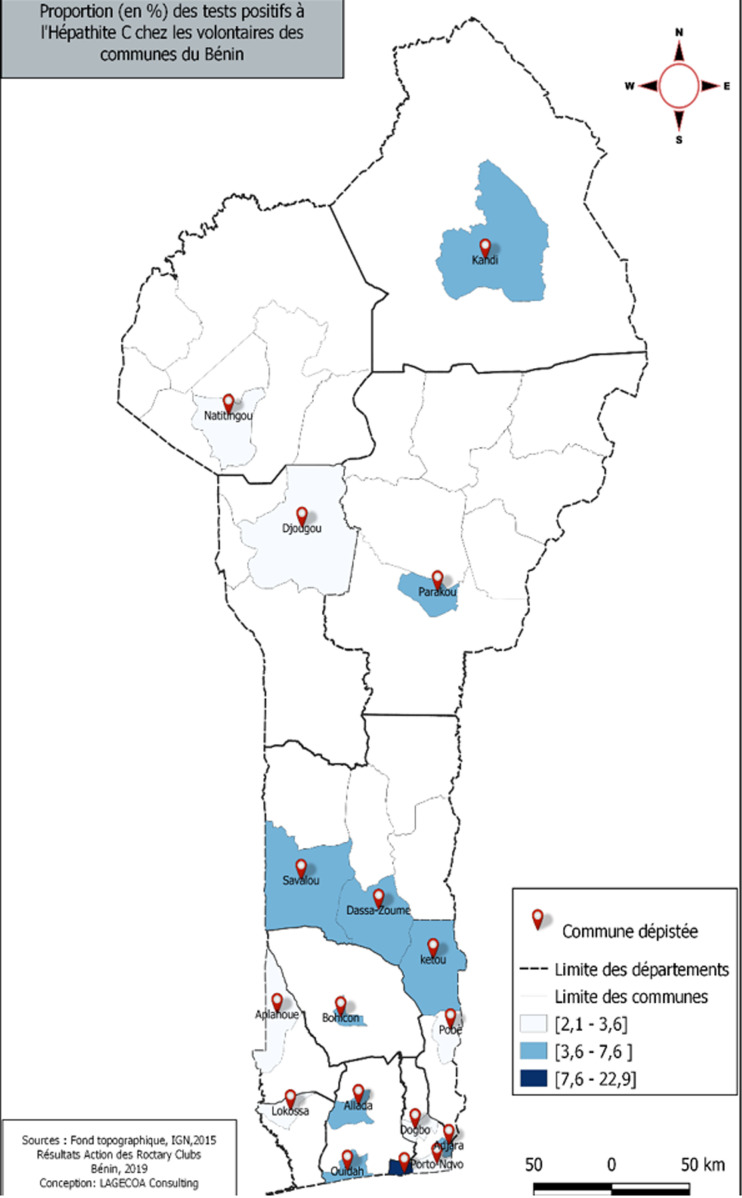
répartition communale des volontaires au test diagnostique rapide (TDR) de l´hépatite B au Bénin: action des Clubs Rotary du Bénin sur Hépatite Zéro en 2019 (n = 9035)

**Collecte de données**: la réalisation de cette opération avait nécessité, une liste initialement établie de sites; répartis en un ou plusieurs postes de dépistage (Figure 1). Chaque club (26 au total au Bénin) avait désigné un point focal ou un gestionnaire de poste de dépistage. Ce dernier était le responsable d´une équipe de dépistage par poste, composée de 03 bénévoles et 03 agents de santé. L´équipe avait évolué selon une procédure pré-établie mise à leur disposition.

**Matériel minimal requis**: chaque poste était doté, d´un cahier d'enregistrement adéquatement renseigné suivant les recommandations du guide d'organisation pratique d'un poste de dépistage, avec quelques données dont: le nom de l´auteur du remplissage du cahier, le numéro d'ordre, les nom et prénom du volontaire et son numéro de téléphone, la profession, l´âge, l´exposition aux risques dans un passé, le résultat d´un éventuel test de dépistage d´hépatite B ou C dans un passé, etc. Un des bénévoles devrait avoir un téléphone portable Android sur lequel était installé l´application Kobocollect qui avait permis de saisir surplace les données, en respectant l´anonymat du sujet. A part les kits de dépistage disponibles, chaque poste avait ses consommables médicaux (alcool, gants, lancettes pour piquer le bout du doigt), une boite de sécurité, un sac poubelle. Tous les cahiers utilisés sur chaque poste étaient retournés au comité national Hépatite Zéro. Des fiches de remise des résultats étaient aussi disponibles. Un rapport journalier sur le nombre de kits utilisés se faisait par le point focal. Pour le dépistage de l´hépatite B, le test rapide d´orientation diagnostique (TROD) utilisé était InTec HBsAg Rapid Test^®^ (InTec Products, Inc; China).

**Procédure de gestion d´un volontaire au test de dépistage**: deux bénévoles au moins se chargeaient de l´accueil et de l´enregistrement du participant pour sa traçabilité dans le cahier préalablement nommé. Il fallait y enregistrer le numéro d´ordre du volontaire et son numéro de téléphone. Avec la fiche de résultat pré remplie, le volontaire au dépistage devait passer chez le 1^er^ agent de santé pour une séance de counseling pré test. Tout de suite après, il passait chez le 2^e^ agent de santé qui lui faisait le test suivant la procédure indiquée, en lui retirant la fiche de résultat. Le résultat du test était apposé sur la fiche de résultats par le 2^e^ agent de santé qui la passait à son tour au 3^e^ agent de santé. A côté de celui-ci se trouvait le bénévole qui avait le portable Android pour administrer le questionnaire au volontaire. A la fin du processus, les résultats étaient donnés au volontaire par l´agent de santé qui était aussi chargé de soumettre l´intéressé à un counseling post test.

**Conduite à tenir face au résultat du dépistage**: en cas de test positif, le sujet était informé par l´agent de santé membre de l´équipe de dépistage. Il le rassurait de ce que, il devra être contacté par le programme national de lutte contre l´hépatite pour des tests de confirmation et une prise en charge. En cas de test négatif, le patient était rassuré qu´il n´a pas l´infection par le VHB ou VHC. Les conseils appropriés sur la prévention de l´infection lui étaient donnés.

**Traitement et analyse des données**: les données issues de cette opération de dépistage avaient permis de mettre en facteur les variables qu´étaient: l´âge, le sexe, la profession, le niveau d´instruction, le lieu de résidence, les antécédents de contact avec le sang (transfusion, scarification), la maladie non transmissible (le diabète). Elles ont été exportées de l´application Kobocollect vers le logiciel Stata pour être analysées selon le processus suivant: une description de la distribution des volontaires au test rapide par le calcul de la moyenne plus ou moins écart type ou de la médiane avec son intervalle inter quartile pour les variables quantitatives, suivi du calcul de proportions des variables qualitatives. L´analyse bivariée des données avait été faite à l´aide du test statistique de Student et du Chi^2^ de Pearson. Elle nous avait permis de déterminer les associations entre le résultat du test rapide et les variables indépendantes. Elle avait également permis de mesurer le rapport de prévalence (RP) et leur intervalle de confiance à 95%. Pour préparer l´analyse multivariée, des variables indicatrices avaient été créées pour les variables ayant des modalités supérieures à deux. De même, certaines modalités de très petits effectifs avaient été fusionnées lorsque c´était nécessaire. Le résultat de l´analyse multivariée avait été obtenu grâce au modèle de régression logistique utilisé pour rechercher les facteurs ajustés et associés au résultat du test de l´hépatite B. Les variables ayant une p-value inférieure ou égale à 20% lors de l´analyse univariée avaient été introduites dans le modèle initial de régression logistique après une élimination pas à pas descendante. Les facteurs qui avaient été retenues dans le modèle final étaient celles dont la p-value était inférieure à 5%. L´adéquation du modèle avait été appréciée avec le test de Hosmer-Lemeshow. Le modèle était dit adéquat quand la p-value était supérieure à 5%.

**Confidentialité**: chaque poste était disposé de manière à ce que l´agent de santé et le bénévole utilisant le téléphone portable Android soient un peu éloigné des autres afin de maintenir la confidentialité des résultats.

**Résultats**

**Caractéristiques de la population étudiée**: au total, nous avions obtenu 9040 dossiers de volontaires au test diagnostique rapide (TDR) de l´hépatite B dont 9035 ont été exploités car cinq (05) dossiers comportaient des données erronées. Les dossiers provenaient de 23 sites de dépistage sur l´ensemble du territoire national (Tableau 1). Environ un volontaire sur quatre (23%) avait effectué son dépistage dans le département du Littoral, seul département multi sites (5 sites); 18,1% et 13,3% respectivement de l´Atlantique et de l´Ouémé. La plupart des volontaires étaient de nationalité Béninoise (99,0%), avec un âge médian de 27 ans (EIQ = 25 ans). La proportion de ceux qui étaient nés avant 2002, l´année de l´introduction du vaccin contre le virus de l´hépatite B, étaient de 28,1% et 22,7% étaient âgés de moins de 15 ans. Plus de la moitié (51,1%) des volontaires étaient célibataires et moins d´un volontaire sur trois (29,7%) étaient mariés. Il s´agissait principalement d´écoliers, élèves ou étudiants (37,9%), de cadres du domaine public ou privé (17,8%) et d´artisans ou ouvriers (11,1%). Un antécédent personnel d´hépatite B était signalé par 333 patients (3,7%).

**Tableau 1:** facteurs associés à la prévalence de l´hépatite B chez les volontaires au test diagnostique rapide (TDR): action des Clubs Rotary du Bénin sur Hépatite Zéro en 2019 (n = 9035)

	Test positif		Test négatif	RP	IC95%	p
	Effectif	%	Effectif			
**Sexe**						
Masculin (n=4664)	364	66,8	4300	1,9	(1,6-2,4)	**0,0000**
Féminin (n=4371)	181	33,2	4190	1		
**Groupes d´âge** (en année)						
Moins de 17 (n=2380)	17	(3,1)	2363	1		
17-27 (n=2260)	133	(24,4)	2127	8,7	(5,2-14,4)	**0,0000**
28-41 (n=2214)	235	(43,1)	1979	16,5	(10,1-27,1)	**0,0000**
42 et plus (n=2181)	160	(29,4)	2021	11,0	(6,7-18,2)	**0,0000**
**Statut matrimonial**						
Célibataires (n=4619)	166	(30,5)	4453	0,4	(0,3-0,5)	**0,0000**
Divorcé(e)/ Veuf (Veuve) (n=353)	24	(4,4)	329	0,8	(0,5-1,2)	0,21
En couple/ Marié(e) (n=4063)	355	(65,1)	3708	1		
**Niveau d´étude**						
Non scolarisé (n=1118)	48	(8,8)	1070	0,5	(0,4-0,7)	**0,0000**
Primaire (n=2053)	78	(14,3)	1975	0,5	(0,4-0,6)	**0,0000**
Secondaire (n=3285)	214	(39,3)	3071	0,8	(0,7-09)	**0,034**
Supérieur (n=2579)	205	(37,6)	2374	1		
**Catégories proféssionnelles**						
Ecoliers/ Elèves/ Etudiants (n=3426)	100	(18,3)	3326	0,3	(0,2-0,4)	**0,0000**
Sans emploi/ Ménagères (n=657)	41	(7,5)	616	0,6	(0,5-0,9)	**0,016**
Artisans/ Ouvriers (n=1005)	82	(15,0)	923	0,9	(0,7-1,1)	**0,31**
Revendeurs/ Commerçants (n=841)	63	(11,6)	778	0,8	(0,6-1,1)	**0,13**
Cadres Public/Privé (n=1608)	150	(27,5)	1458	1		
Retraités/ Autres (n=1498)	109	(20,0)	1389	0,8	(0,6-0,9)	**0,038**
**Diabètique**						
Oui (n=223)	21	(3,9)	202	1,6	(1,1-2,6)	**0,031**
Non (n=8812)	524	(96,1)	8288	1		
**ATCD de transfusion sanguine**						
Oui (n=394)	31	(5,7)	363	1,4	(0,9-1,9)	**0,12**
Non (n=8641)	514	(94,3)	8127	1		
**ATCD de scarification**						
Oui (n=2428)	184	(33,8)	2244	1,4	(1,2-1,7)	**0,0002**
Non (n=6607)	361	(66,2)	6246	1		
**ATCD d´hépatite C**						
Oui (n=101)	11	(2,0)	90	1,9	(1,1-3,6)	**0,04**
Non (n=8934)	534	(98,0)	8400	1		

* RP = rapport de prevalence

**Séroprévalence de l´hépatite B**: selon les résultats du TROD de l'HVB, les volontaires testés positifs étaient au nombre de 545 sur les 9035, soit une prévalence de 6,0% [IC95%=5, 5%-6,5%]. En fonction de l´âge, en prenant pour référence l´an 2002 où le vaccin contre l´hépatite B avait été introduit dans le PEV au Bénin, pour les sujets nés avant 2002, le test du VHB était positif chez 527/6496 (soit une séroprévalence de 8,1%) tandis que pour ceux nés à partir de 2002, il était positif chez 18/2539 (soit 0,7%).

**Facteurs associés au portage de l´Ag HBs**: en analyse univariée, l´âge apparaît associé au portage du VHB. La comparaison des moyennes d´âge montre que les volontaires positifs au test paraissaient plus âgés que les autres (35,5 ± 12,3 ans versus 28,9 ± 17,7 ans; p<0,0001). Par ailleurs, les chez les sujets de moins de 17 ans (nés avant 2002), la séroprévalence du VHB était de 8,1% (527/6496) tandis qu´elle était de 0,7% (18/2539) chez les plus de 17 ans, avec une différence statistiquement significative (p<0,0001). Les sujets de 17 ans ou plus étaient 8,7 à 16,5 fois plus touchés par le VHB que ceux de moins de 17 ans (Tableau 1). En dehors de l´âge, les autres facteurs associés à l´analyse univariée étaient (Tableau 1) le sexe (hommes 1,9 fois plus de risque d´être touchés que les femmes), p<0,0001; b) le statut matrimonial (les célibataires moins touchés que les autres), p<0,0001; c) le niveau d´étude (les sujets non scolarisés ou de niveau primaire (p<0,0001) et ceux de niveau secondaire (p=0,034) moins touchés par le VHB que ceux de niveau supérieur ou universitaire); d) la profession (les écoliers/élèves/étudiants (p<0,0001), e) les sans-emploi/ménagères (p=0,016), et les retraités (p=0,038) moins touchés par le VHB que les autres catégories professionnelles); f) l´antécédent de diabète (les diabétiques étaient 1,6 fois plus à risque d´être touchés par le VHB que les non diabétiques), p=0,038; g) la notion de scarification (1,4 fois plus de risque d´être touchés en cas de scarification), p=0,0002; h) et l´antécédent d´hépatite C (1,9 fois plus de risque d´HVB en cas d´antécédent d´hépatite C), p=0,04.

En analyse multivariée, le sexe, le niveau d´étude et l´âge étaient les seuls facteurs indépendamment associés au portage de l´Ag HBs (Tableau 2). Connaissant le niveau d´étude et l´âge, les individus de sexe masculin étaient deux fois plus à risque de porter le VHB que les ceux du sexe féminin. Concernant l´âge, les volontiers âgés de plus de 17 ans étaient plus à risque de porter le VHB. De façon spécifique, à sexe et niveau d´étude connu, les volontiers de 17 à 27 ans, 28 à 41 ans et de 42 ans et plus étaient respectivement 9 fois, 17 fois et 11 fois plus à risque de porter le VHB que ceux de moins de 17 ans. Enfin, les volontaires ayant un niveau d´étude secondaire étaient 1,2 fois plus à risque d´être porteur du VHB que ceux d´un niveau supérieur (universitaire), en considérant l´âge et le sexe.

**Tableau 2:** analyse multivariée des facteurs associés à la survenue du virus de l´hépatite B chez les volontaires au test diagnostique rapide au Bénin en 2019 (n = 9035)

Variables retenues dans le modèle	RPaj	IC95%	p
**Sexe**			
Masculin	1,9	1,6-2,3	**0,000**
Féminin	1		
**Niveau d´étude**			
Non scolarisé	1,3	0,7-1,4	0,98
Primaire	1,2	0,9-1,6	0,22
Secondaire	1,2	1,1-1,5	**0,04**
Supérieur	1		
**Catégories d´âge selon les quartiles**			
Moins de 17	1		
17-27	9,3	5,5-15,8	**0,000**
28-41	17,2	10,3-28,6	**0,000**
42 et plus	11,1	6,7-18,6	**0,000**

Test de Hosmer-Lemeshow : Chi2=5,96 (p=0,55)

## Discussion

De cette étude, il ressort que la séroprévalence de l´hépatite B est de 6%. Ceci classe le Bénin parmi les pays d´endémicité intermédiaire pour le VHB (entre 2 et 7%). Cette prévalence est plus faible que les 9,9% rapportés comme moyenne nationale chez les nouveaux donneurs de sang dans les centres de transfusion sanguine en 2013 [6]. Elle est aussi plus faible que celle de 14% trouvée en 2017 chez les gestantes à Cotonou [10] et à Parakou [11]. Ces différences peuvent s´expliquer par le fait que notre étude a inclus un bon nombre des sujets jeunes âgés de moins de 18 ans, ce qui n´est pas le cas pour les études faites chez les gestantes ou les centres de transfusion sanguine. En effet, 28,1% des sujets inclus étaient âgés de moins de 18 ans. Et la prévalence de l´hépatite B dans cette tranche d´âge était faible (0,7%) contrairement aux sujets de 17 ans et plus (8,1%). Rappelons que depuis 2002, le vaccin contre l´hépatite B a été introduit dans le PEV, ce qui a donc contribué à une nette diminution de la fréquence de l´HVB chez les sujets de moins de 17 ans. Ce constat a été aussi fait en Gambie en 2015 chez les gestantes: la séroprévalence du VHB était de 13,7% chez les gestantes n´ayant jamais été vaccinés contre le VHB versus 2,3% chez celles déjà vaccinées [12]. Ainsi, en ajoutant une dose dans les 24 premières heures de vie, notre pays peut-il espérer atteindre l´élimination de l´hépatite B à l´horizon 2030, telle que souhaitée par l´OMS, en parvenant à réduire de 90% les nouvelles infections au VHB et de 65% les décès liés au VHB [13].

En comparaison à d´autres pays africains, au Burkina Faso, en milieu rural au Burkina Faso en 2018, il avait été rapporté des prévalences semblables de 6,3% en général et 0,8% chez les enfants [14]. Au Togo, chez 1213 sujets dépistés à Lomé pour les hépatites B et C à l´occasion de la journée mondiale contre l´hépatite, une prévalence élevée de 16,36% avait été trouvée pour l´HVB [15]. Au Ghana, une revue systématique sur la période de 2015-2019 une prévalence de 8,36% chez l´adulte, 14,3% chez les adolescents et 0,55% chez les enfants de moins de 5 ans [16]. Une prévalence plus faible, de 4,6%, a été rapportée en 2002 au Burundi en population générale [17]. Au Maghreb, des prévalences varient selon les pays [18]: 1 à 2,35% au Maroc, 4 à 7% en Tunisie, 1,6 à 3,6% en Algérie, 1,3 à 5,8% en Lybie et 16,8 à 22% en Mauritanie.

Concernant les facteurs associés dans notre travail, plusieurs étaient trouvés en analyse univariée: l´âge, le sexe, le statut matrimonial, le niveau d´étude, la profession et les antécédents de diabète, de scarification et d´hépatite C. Cependant, ceux qui ressortaient en analyse multivariée étaient l´âge, le sexe et le niveau d´étude. Dans l´étude faite au Burkina en milieu rural, les facteurs associés étaient l´âge, le sexe et l´antécédent de scarification [14]. Une étude à Bouaké, avait relevé comme facteurs associés le sexe, l´âge, le statut vaccinal et l´antécédent de transfusion sanguine [19]. L´étude au Burundi en 2002 avait aussi rapporté le sexe comme facteur associé [17]. En Ethiopie, seul l´asthénie et la fatigue étaient associées à une infection au VHB [20]. Dans une étude chez des militaires sénégalais envoyés en mission au Darfour, les facteurs associés à l´hépatite B étaient: l´âge jeune, le niveau d´étude universitaire, et l´exposition sexuelle [21]. La fréquence plus élevée du VHB chez les hommes est constamment rapportée dans la littérature [21-23]. Elle peut s´expliquer par de facteurs génétiques favorisant la persistance du virus chez les hommes [24]. Des facteurs hormonaux pourraient intervenir pour favoriser que les femmes éliminent plus le VHB expliquant la différence entre les deux sexes [25]. Quant à la différence liée à l´âge, elle s´explique surtout par la vaccination anti-VHB qui avait été systématique chez les sujets de moins de 17 ans, ce qui n´était pas le cas chez les 17 ans et plus. Ce bénéfice de la vaccination sur la réduction de la prévalence de l´hépatite B a été rapporté dans plusieurs pays [12,22]. La principale limite de la présente étude réside dans l´échantillonnage. En effet, elle a porté sur des volontaires au test sur toute l´étendue du territoire national. Les sites de dépistage étant souvent logés dans des universités ou écoles, l´étude avait inclus une majorité d´apprenants, ce qui pourrait constituer un biais de recrutement. Les résultats obtenus ne peuvent donc pas être généralisés à tous les profils du fait que la sélection n´avait pas été aléatoire.

## Conclusion

La séroprévalence du VHB est de 6% dans la population étudiée. L´infection par le VHB semble toucher les sujets de sexe masculin, âgés de plus de 17 ans, et ayant un niveau d´étude secondaire. Toutefois, l´étude ayant porté sur des volontaires au dépistage, ces données nécessitent d´être confirmées par un travail d´envergure nationale avec un recrutement aléatoire afin de mieux connaître l´ampleur actuelle de l´hépatite B au Bénin.

### Etat des connaissances sur le sujet

- L´hépatite B est fréquente au Bénin (9,9%) d´après des données nationales en 2013 chez les nouveaux donneurs de sang;
- Le vaccin contre l´hépatite B fait partie du Programme élargi de vaccination depuis 2002 au Bénin et est administré à tous les nourrissons dès la 6^e^ semaine de vie.

### Contribution de notre étude à la connaissance

- La prévalence de l´hépatite B en population générale à Cotonou semble en diminution, et s´élève à 6%;
- La vaccination contre l´hépatite B introduite en 2002 a probablement contribué à cette diminution (séroprévalence du VHB à 0,7% chez les sujets nés après 2002 versus 8,1% chez ceux nés avant);
- Les facteurs associés à l´infection au VHB sont: l´âge, le sexe et le niveau d´étude.

## Remerciements

C´est le lieu de remercier les 26 Clubs du Rotary du Bénin qui ont pu mobiliser des ressources pour la réalisation et la réussite de cette action « Hépatite Zéro » du Rotary International. Nous ne pouvons non plus oublier les vaillants volontaires qui y ont contribué.
